# “The double-edged sword of inflated help”: Unravelling the motivation crowding in community question-answering platforms

**DOI:** 10.1371/journal.pone.0297627

**Published:** 2024-03-13

**Authors:** Vaibhav Krishna, Yash Raj Shrestha, Georg von Krogh

**Affiliations:** 1 Yale Institute of Network Science, Yale University, New Haven, Connecticut, United States of America; 2 Department of Sociology, Yale University, New Haven, Connecticut, United States of America; 3 Faculty of Business and Economics (HEC), University of Lausanne, Lausanne, Switzerland; 4 Department of Management, Technology and Economics, ETH Zürich, Zürich, Switzerland; 5 ETH AI Center, ETH Zürich, Zürich, Switzerland; IRCCS Istituto Ortopedico Rizzoli, ITALY

## Abstract

The growth of digital platforms has led to the proliferation of Online Communities, providing individuals with opportunities to seek help and share knowledge. A key challenge of help-related platforms that address technical questions (i.e., utilitarian, rather than opinion or supportive) is to ensure the contributions address seekers’ specific information needs. Despite growing academic interest in such platforms, research has mainly focused on factors that influence the quantity of contributions, ignoring whether these contributions effectively helped the seekers. To fill this research gap, this study draws upon theories of self-determination and motivation crowding to examine contributing behaviors that result in successful helping. By analyzing a rich dataset collected from an online Q&A platform, we find that gains in a help provider’s past rewards positively influence the success of contribution. Further, while previous studies suggest that external rewards result in a high quantity of contribution, our findings show that an inflated frequency of contribution leads to a crowding-out effect. Specifically, the contribution frequency has a curvilinear relationship with the success of the contribution. Taken together, these findings demonstrate there is a need to revisit the gamification mechanism on help-related platforms to ensure the success of knowledge contribution. This is crucial for the sustainability of these platforms as low-quality answers can lead users to mistrust and eventually leave the platform.

## 1. Introduction

Help-seeking is an important activity often taken by individuals in the face of complex situations that they do not fully understand [1–4]. Confronted with doubts, individuals often turn to other trusted individuals for advice, an external source of knowledge (such as books or magazines), or other conventional channels (such as media) for information, help, or advice. Lately, the advancements in digital platforms have brought a paradigm shift in how individuals interact with each other and share and acquire information. The proliferation of online communities (such as Github, Wikipedia, and Quora) is an outcome of this paradigm shift which has drawn great interest in understanding how these platforms foster interpersonal information sharing and knowledge exchange [5]. With the popularity of digital platforms and online communities, individuals increasingly turn to the internet to seek help and advice [6]. The importance of these communities was accentuated during the COVID-19 pandemic which substantially increased the demand for online information exchange [7].

Online question-and-answer communities (CQA) are web-based services that have grown rapidly as they facilitate the exchange of customized information and knowledge [8]. The operation of these communities requires the assembly of a critical mass of seekers who are willing to seek help as well as contributors who are willing to share their knowledge [9]. The presence of help seekers and providers instantiates a reinforcing cycle where seekers’ seeking activity motivates contributors to provide help, and the perceived likelihood of finding help reinforces seekers’ seeking behavior. Despite the ease of access to a diverse and large set of help providers, the variance in the quality of knowledge contributed on these platforms can pose a significant challenge to effectively satisfying the seekers’ specific information needs [10]. Low-quality or incomplete answers could lead seekers to distrust or leave the community, posing a challenge to the sustainability of these communities [11]. As a result, maintaining steady and high-quality help is crucial to the very survival of such communities.

Cumulated research on online communities (OCs) has shed light on the phenomenon of helping behavior on digital platforms, focusing mainly on the motivation of contributors to provide help [12–14]. Yet past studies have primarily focused on the quantity of contributions (that is, the frequency of help by providers), while the success of knowledge contribution remains weakly understood. In the literature, various motivational factors explain the quantity of contributions. In many of these studies, intrinsic as well as extrinsic motivations (in terms of rewards) are emphasized as important factors that may influence and promote contributions. However, despite the reinforcing influence of external rewards on the frequency of contributions, scholars [15, 16] have indicated that extrinsic rewards can lead to reduced effort in the activity in the long run. This is referred to as the motivation crowding effect where extrinsic intervention can undermine the intrinsic motivation required for the activity [17]. Thus, while the external rewards may encourage the contributors to increase the frequency of contribution, the quality of contribution is not guaranteed.

In our study, we explore the knowledge-sharing activity in online CQA to investigate what shapes effective and successful knowledge contribution on these platforms. Drawing on theories on self-determination and motivation crowding out, this study examines the behaviors of contributors that influence the success of answers produced in these communities. While the quantity aspect of knowledge contribution in OCs is widely studied [18–20], this study contributes to online community literature by providing an enhanced understanding of the success of knowledge contribution. Further, this study advanced our understanding of motivation crowding literature and provides empirical evidence in the CQA communities that are largely governed by pro-social behavior. While the crowding-out effect is explored in communities that are based on monetary incentives [21, 22] its effect in communities that do not contain any tangible reward system to motivate participants is less known.

The rest of this paper is organized as follows. In Section 2 we review the literature related to knowledge-sharing and motivations that influence individuals’ knowledge-sharing behavior. In Section 3, we elaborate on our research model and formulate hypotheses. We provide the research method in Section 4. In Section 5, we present the data analysis and results. We conclude the paper in Section 6.

## 2. Theoretical background

OCs have been studied in a variety of contexts that include problem-solving [23, 24], innovation [25–27], and knowledge sharing [28, 29]. The knowledge-sharing and contribution in OCs have drawn considerable attention from scholars. To understand the social mechanism and individual differences that impact knowledge contributions on these platforms, researchers have applied various theories such as social capital theory [12, 29], self-determination theory [30, 31], social exchange theory [5, 12, 32], goal setting theories [33], and others.

CQA is a popular type of OC that facilitates knowledge exchange in the form of asking and answering questions [18]. What sets CQAs apart from other OCs is their focus on issue-oriented exchanges, rather than building relationships between community members [34]. The knowledge exchange on CQAs starts with a help seeker posting a question on the platform and subsequently receiving answers from other members. The seeker then chooses the answer which provides the complete information the seeker is looking for as the best answer. Therefore, the process of knowledge exchange in CQAs limits the room for social interaction and is primarily driven by individuals’ personal needs. As a result, the help seekers and providers in CQAs tend to focus on knowledge exchange, with minimal socializing and relationship building.

The majority of extant studies on OCs, including the CQAs, have focused on the quantity aspect of knowledge contribution, i.e., the users’ behavioral frequency of contributing knowledge and helping [19, 20, 32]. Few studies have examined the quality aspect of knowledge contribution in OCs by leveraging the social capital perspective [29, 35]. Furthermore, there exists a corpus of technical research focused on developing algorithms aimed at identifying the best answers among those already posted. Additionally, this research also delves into the identification of the most proficient answerers from the pool of available community members who possess the capability to provide high-quality answers to specific questions [36, 37].

In the context of sustaining OCs, both quantity and quality play pivotal roles. However, within CQA platforms, the emphasis on quality is heightened even further. This stems from the fact that answers must not only exhibit superior quality but also be finely tailored to precisely address the unique needs of the seeker. Consequently, contributing answers on these platforms demands contributors to invest a distinctive level of cognitive effort. This diligence is pivotal in ensuring that their responses impeccably align with the seekers’ prerequisites for relevance and accuracy [13]. As a result, seekers often designate such answers as the best or most successful solutions.

In order to examine individuals’ contributing behavior that leads to success, we draw on self-determination theory (SDT) [38], [39] and motivation crowding theory (MCT) [15, 16]. SDT has been widely used to understand knowledge contribution behavior in various knowledge-sharing platforms like electronic knowledge repositories, open-source software communities, and online discussion forums [30, 31].

SDT suggests that human behavior can be explained by three distinct types of motivation: intrinsic motivation, internalized extrinsic motivation, and external regulation [40]. Intrinsic motivation (e.g., *knowledge self-efficacy, enjoying helping others [5, 41])* represents the most prototypic instance of self-determination as it is derived from the inherent satisfaction of performing the behavior per se [38]. External regulation involves using incentives (*e.g., rewards [42]*) to reinforce desired behavior. Finally, internalized extrinsic motivation is the motivation that comes from external influences at first and then becomes internalized as one’s own (*e.g., learning [43, 44]*) [39].

According to SDT, the level of self-determination that stems from various motivations can result in differing behavioral outcomes through unique regulation processes. Self-determined motivations, characterized by a strong drive towards novelty and challenge, align with the positive aspect of human nature that fosters curiosity, creativity, and increased involvement, resulting in improved performance outcomes [40]. In the case of learning, while help seekers directly engage in learning through seeking help and gaining knowledge from others, help providers do not inherently engage in learning. Instead, they experience learning as a by-product of helping others, as they delve into uncharted territories or revisit previously acquired knowledge [20]. Finally, while extrinsic motivations are effective in promoting contributing behavior, the quality of the contributions is not guaranteed [15]. When behavior is reinforced by an external source, temporary compliance can be achieved, but the inherent interest in the behavior will be diminished [45]. Thus, when motivated by receiving an external reward, individuals might artificially inflate their behavioral frequency at the cost of quality [46].

This idea aligns with the *motivation crowding effect* documented in the literature [15, 16]. It suggests that the presence of external rewards can diminish intrinsic motivation, which results in decreased effort in the related activity. While motivation crowding theory (MCT) has been studied in open-source projects [42, 47, 48], its role in knowledge-sharing platforms such as CQAs has yet to be explored. Nonetheless, it is precisely within these communities that the introduction of external rewards might prompt individuals to increase the frequency with which they answer questions. This, however, could potentially lead to a decreased emphasis on the meticulous details necessary for adequately addressing the seekers’ specific information requirements.

Various studies in psychology and economics show that under specific conditions there is a trade-off between extrinsic rewards and intrinsic motivation. Introducing extrinsic rewards for doing an intrinsically interesting activity can have a negative effect in the long run. It is seen that people tend to feel controlled by rewards, engendering a shift in the benefits from intrinsic to extrinsic. For example, Osterloh and Frey, (2000) [49] in their studying found that while motivating children with rewards for doing their homework is successful in the short run, in the long term this leads to a lower willingness of the children to do their homework without the reward. Similarly, Gneezy and Rustichini (2000) [50] have shown that adding a small monetary compensation can decrease high school students’ effort in volunteer work.

Scholars argue that extrinsic incentives can shift individuals’ focus from altruistic motivation to rewards leading to motivational crowding-out and subsequent worse performance [17, 51]. The crowding-out effect has been studied in product review platforms where consumers are sometimes paid or receive gifts for writing reviews. Studies show that while these extrinsic rewards motivate users to write more reviews, the reviews on average are shorter in length [52] and might contain more biased opinions [21, 22]. Therefore, while CQA studies show rewards promote a high quantity of knowledge sharing, it is interesting to examine the crowding effect that may exist in these communities.

SDT and MCT provide an appropriate theoretical lens to address our research question for two major reasons. First, the issue-oriented knowledge contribution in CQA makes these communities individually driven rather than socially oriented. Thus, we believe SDT is prevalent given its focus on motivational factors that relate to individuals’ activities. Second, the literature shows what motivates individuals to contribute more on these platforms. Given contributors on CQA platforms have to make a significant effort to tailor their answers to the needs of the seekers [13], it is important to understand if the high frequency of contribution in CQA platforms has a crowding-out effect.

## 3. Research model and hypothesis

**Fig 1:**
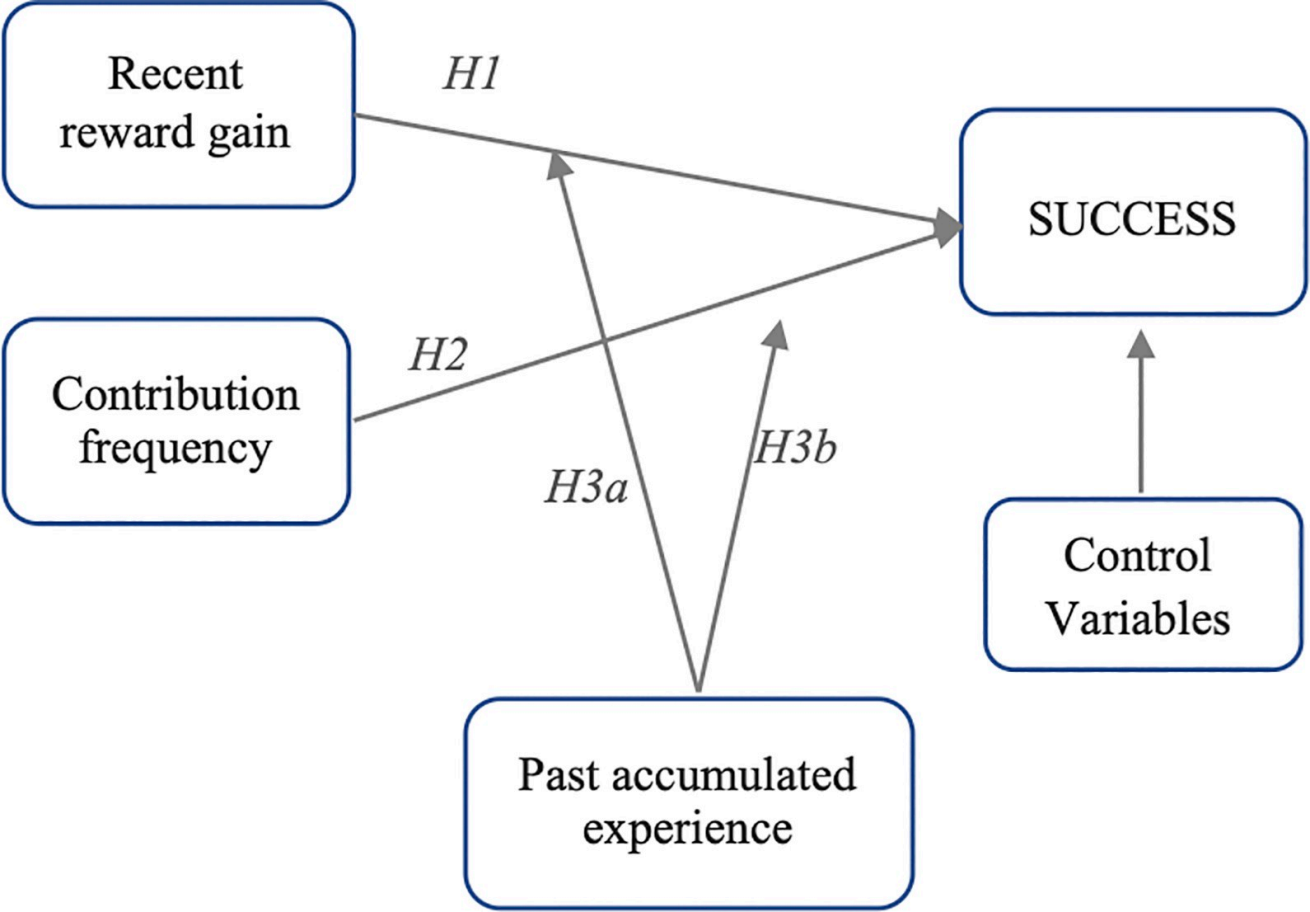
A theoretical model based on SDT and MCT.

We depict the proposed theoretical model in Fig 1.

First, we examine the main effects of recent reward gains and the contribution frequency on the success of knowledge contribution. Extrinsic regulation in organizational setting literature is attributed to the motive to attain better compensation, higher job security, and promotion [9, 53, 54]. Similarly, in the context of OCs such as OSS communities, the monetary rewards developers get for their intellectual inputs are found to be effective in promoting the frequency of knowledge contribution behavior [5, 9]. Such rewards are not available in CQAs. CQA platforms are designed based on gamified settings where knowledge contributors are incentivized in different forms such as levels, scores, badges, etc. The scores users receive for their contributions on the platform serve as a reward and play a similar role as the tangible rewards in other settings. Users in these systems accumulate these scores in a reputation system that highlights their engagement and the trust of the community members.

The scores add to the cumulative reputation score which improves help-providers ranking in the community and unlocks certain privileges for high-rank users. These digital platforms offer contributors the chance to showcase their verified and credible expertise, which can be included on their resumes for job-hunting purposes. Indeed, IT companies and recruiters often check profiles on these platforms when searching for potential hires [55]. In this way, the ranking and access to privileges reinforce further contributions on the platforms. Based on SDT, help providers can be motivated to put more effort into providing high-quality answers to increase their reputation score on the platform. Further, positive feedback is associated with enhanced interest, persistence, and cognitive investment in the focal activity [56], which leads to better performance. Given the availability bias in human decision-making which states that people tend to weigh more recent events [57], we believe that the effect of recent positive feedback would be more significant.

Therefore, we propose that:

**H1:** *Participants who have received higher recent positive feedback will put more effort into contributing high-quality answers*, *thus increasing the likelihood of success*.

In CQA communities, as contributors engage in providing help, they also experience learning. Learning refers to the belief that individuals can benefit from self-learning by contributing knowledge either by exercising existing knowledge or exploring unknown areas [20, 43]. Individuals who have a strong desire to learn from their experience need to review what they already know or extend their knowledge base when formulating an answer [19]. Thus, as individuals participate more in contributing answers, they gain a better understanding of articulating their answers effectively and concisely.

However, inflated contributing behavior driven by a desire for external rewards could also result in crowding out intrinsically motivated activities [58, 59]. Within the realm of CQAs, providing high-quality answers entails various cognitive efforts from the contributors. As the contributors are expected to understand the context in which the help is required, they need to put effort into formulating an answer that matches the expectation of the seeker in terms of relevance and accuracy [13]. However, contributors driven by external incentives may overlook the depth and thoroughness of their responses when submitting a substantial number of answers within a short span of time. Hence, the challenge of receiving low-quality answers increases in the case of individuals artificially inflate their contribution frequency.

Therefore, when considering both factors, while increased participation in answer writing may lead to improved answer quality due to a learning effect, the excessive frequency of contributions due to the crowding-out effect would likely have a negative impact on the quality of answers. Thus, based on the learning and the crowding-out effect, we propose:

**H2:** *The contribution frequency has an inverted U-shaped curvilinear relationship with the success of knowledge contribution*.

Next, we expect a moderating effect of the past accumulated experience of contributors. Previous research has demonstrated that the accumulation of experience significantly shapes individuals’ perceptions and attitudes toward virtual communities [60, 61]. As members engage more with a community over time, their motivations, values, and attitudes tend to evolve. This perspective has also been underscored in prior studies, emphasizing that the dominance of motivation varies in conjunction with the depth of contribution [62]. Notably, studies have revealed that the factors influencing members’ decisions to contribute knowledge to a virtual community differ from their initial knowledge contributions. Participants in virtual communities are driven to share knowledge for multifarious reasons, yet their preferences may diverge based on their experience. Therefore, considering members with distinct past contribution experiences, it is reasonable to assume that different motivations may exert distinct influences on an individual’s contribution behavior. Accordingly, we posit that an enhanced contribution experience could alter one’s preferences, leading us to hypothesize that the influence of various factors may evolve in tandem with individuals’ accumulated experience. Thus, while positive feedback or rewards possess the potential to shape participation behaviors, guiding the focus toward task accomplishment, however, the degree of this attention shift varies according to different levels of experience. For example, existing studies have illuminated that novices who receive feedback are more inclined to contribute content in subsequent instances [63]. Notably positive feedback is proven to wield greater significance in augmenting the subsequent quality of content contributed by those with less experience. Such positive feedback not only offers encouragement but also provides guidance, fostering enhanced confidence for future task performance. Nonetheless, as contributors amass more experience, the symbolic nature of positive feedback could potentially lose its informativeness. Comparable to findings from previous research on supervisor feedback, proficient individuals show decreased responsiveness to positive supervisory feedback due to their already attained expertise [64]. Therefore, we posit:

**H3a**: *Past contributing experience diminishes the positive effect of rewards on the success of knowledge contribution such that the effect will be less positive for more experienced users*.

The influence of past accumulated experience may also moderate the impact of contribution frequency on answer success. In terms of the learning effect stemming from contribution frequency, we anticipate that individuals with limited experience can derive substantial benefits from composing more answers. According to the motivation-capability perspective [65], domain-specific skills hold an equal significance for performance as motivation does. Capability is expected to grow in tandem with experience, which is an outcome of repetitive engagement in a task, gaining exposure, or fostering a heightened degree of familiarity with specific domains [66]. Within the context of CQAs, experience pertains to an individual’s history of writing answers within the community. Hence, novices have the opportunity to enhance their proficiency in formulating answers that align with the information requirements of seekers. In contrast, learning gains in experienced users tend to be marginal, given their heightened perception of competence and skills. Regarding the crowding-out effect, the quality and success of answers can suffer adverse repercussions due to an inflated frequency of contributions across all users. In summary, we propose:

**H3b**: *Past accumulated experience renders the relationship between contribution frequency and success in an inverted U-shaped curvilinear manner*, *essentially making it monotonous*. *In simpler terms*, *for experienced contributors*, *while the positive impact of learning remains marginal at the onset*, *the crowding-out effect diminishes the likelihood of success with increased contribution frequency*. *For users with less experience*, *the learning effect predominates over the crowding-out effect initially*, *enhancing the likelihood of success*. *However*, *as contribution frequency increases*, *the negative impact of crowding out surpasses the positive effect of learning*.

## 4. Data and method

We chose Stack Exchange (SE), a popular CQA platform for examining the help-seeking process. Stack Exchange is a network of more than 170 diverse question-answering sites, each “created and run by experts and enthusiasts, who are passionate about a specific topic” and provide “answers to practical, detailed questions”. Stack Exchange communities provide participants with a platform where they can gain valuable information and seek help from motivated experts with whom they can engage. SE provides participants with an open environment where anyone can post questions, and others can provide answers to the posted questions for free. SE started with the Stack Overflow Q&A community for computer programmers in 2008 and expanded into a network of different communities primarily focusing on technology but also other domains, like science, arts, and business. As of Dec 2020, with 5 billion visits and 10 billion page views, SE is one of the most popular CQA web destinations (*https*:*//stackexchange*.*com/about*).

### Research setting

We examine the data based on the recent publicly available data archive (*https*:*//archive*.*org/download/stackexchange*) provided by the SE in December 2020. We studied the ‘Super User’ CQA network (*https*:*//superuser*.*com*) which is a focused community on topics related to computer users and developers. The downloaded dataset for each site included user, post, and vote information; the user information contains a unique identifier for each user, their tenure, and reputation score as of December 2020; the post information includes post type (question or answer), post author, time of post, post body in raw text format, and the number of votes received; vote information contains vote date, vote type (upvote, downvote, etc.), and the corresponding post being voted. In sum, we are able to get access to unique data that captured fine-grained, time-stamped users’ interacting behavior related to various activities on the SE platform. To answer our research question, we collect question-answer pairs and the activity around them. In our context, the instance of help-seeking starts once a seeker posts a question on the SE platform. The question attracts attention from the community where others provide potential answers. Based on the answers received to the question, the seeker marks one of the answers as an “accepted answer”. Stack Exchange indicates a green tick symbol adjacent to the answer that received the accepted vote from the seeker and displays the following message: “The question owner accepted this as the best answer”.

### Data sampling and variables

Given the highly dynamic nature of online platforms [67], we use the most recent data (Jan 2017-Dec 2019) to examine our hypotheses. We filtered the questions that did not receive any answers from our dataset. The final dataset comprises 55,725 help-seeking instances posted by 40,268 different seekers for which 17,320 unique contributors provided 72959 answers. From the answers, 22,865 answers received the accepted vote from the seekers, resulting in a ~31% success rate.

Next, we draw from previous studies to measure variables and make some adjustments to better suit the context of the target community.

### Dependent variable

Different measures have been used in previous studies to assess the quality of contribution on knowledge platforms. This includes manual content analysis [12], survey [13], or using the votes received on the answers [6] by other members on the platforms. On the SE platform, the seeker can mark one of the answers as an “accepted answer”. In our context as we focus on measuring the success of seeking activity, the “accepted answer” indicator seems appropriate, given that question-askers are in the best position to determine if their queries have been answered to their satisfaction. Thus, we coded the dependent variable–‘Success’ as “1” if the seeker marked one of the answers received as “accepted”, otherwise it was marked as “0”.

### Independent variables

Contributors on CQA platforms receive feedback in the form of votes on their answers from other members of the community [31]. The recognition in the form of votes received on the contribution signifies the contributor’s perception that the contribution received attention or acknowledgment from the other members of the community [32, 68]. Positive feedback acts as a motivator for contributors, encouraging the continuation of high-quality contributions. Additionally, we believe that the effect of recent positive feedback would be more significant, aligning with the availability bias observed in human decision-making, wherein recent events hold greater influence [57]. Furthermore, contributors who receive equivalent rewards for fewer contributions may be motivated differently compared to those requiring more contributions to attain the same reward. Therefore, to isolate the impact of reward gain, we utilized the average recent votes per answer received by the contributors in the previous week as a metric.

Motivated by extrinsic rewards, the contributors on the CQA platforms may intensify their contribution. Although heightened platform engagement brings about some additional learning for contributors, an excessively frequent contribution pattern can lead to a crowding-out effect [46]. This suggests that contributors, driven by external incentives, may overlook the depth and thoroughness of their responses when submitting a substantial number of answers within a short span of time. To quantify this, we assess contributors’ contribution frequency by considering the number of answers posted in the previous week.

Past accumulated experience pertains to the historical record of answer contributions on the CQA platform. Contributors who engage actively on the platform and possess a track record of furnishing beneficial answers tend to nurture a higher level of confidence in their capacity to offer valuable knowledge to the community [12, 69]. Previous studies have underscored that past experience stands as one of the most robust predictors of future behaviors. As a result, we compute the cumulative count of answers contributed by a user up until the point of the answering in the specific question-answer pair, serving as a measure of past accumulated experience.

### Control variables

To account for the influence of other factors on the success of the answer, we incorporate several control variables derived from existing helping and online community literature [69–72]. These variables play a crucial role in refining our model and ensuring that the observed effects are not confounded by external factors. In particular, we account for factors that include the *seeker’s reputation*, *question length*, *answer characteristics*, and *time to answer*.

Existing research on helping and knowledge sharing underscores that individuals within groups actively seek to diminish interpersonal status differences [73, 74]. As individuals tend to be held in higher esteem if they appear to give more or superior help to others, the social exchange behavior of helping can confer social status to them [70]. Consequently, the act of helping, as a form of social exchange, becomes a means for individuals to attain or enhance their social status within a group [75]. Specifically, they may try to impress those with higher status to obtain recognition [76] and dedicate more effort to exchanging knowledge. In CQA, individuals collect reputation scores that signal their expertise. This *reputation score*, which appears as user profile information below the question posted by the seeker, signals the social status of the seeker to the community members. Thus, a request from a high-reputation score seeker creates an opportunity to attract attention from other members striving to improve their relative ranking. We measure the reputation score of the seeker at the time of question using the number of votes the seeker received in the past and the metrics calculation as given on the stack-exchange webpage (*https*:*//stackoverflow*.*com/help/whats-reputation*).

Prior literature on OCs has underscored that problems that stand out compared to alternative targets are more likely to attract attention [72]. Detailed questions are posited to attract more contributors, indicating a higher likelihood of success from an attention perspective. Longer, more detailed questions can be more engaging as the contributor gets drawn into the details, as well as dominate the provider’s field of vision on screen crowing out other stimuli [77]. Thus, *question length* is likely to affect the quality of knowledge exchange by a contributor. We measure the number of words in the question post to measure the length.

Extant research on online communities has demonstrated that the determinants of success in online question answering encompass presentation quality that aligns with the community standards [71]. Extensive studies have been conducted, employing discriminative features and classifiers to identify the best answers. For example, earlier works have indicated that answer length prevails as a dominant feature among others in identifying the best answer [78]. The hallmark of a high-quality answer encompasses traits like clarity, visual adequacy, and the provision of necessary references and citations to external resources. Unique structural attributes such as source code and external references [79] have been recognized as influential predictors of high-quality answers within technical CQAs. On Stack Exchange, simple textual metrics are usually used to pre-filter low-quality posts which include the length of the answers, the use of links, as well as the presence of code snippets for technical questions. We measure answer characteristics such as *answer length* by counting the number of words in the answer, identify the presence of *external links* through the number of URLs, and denote the presence of a *code snippet* using a binary variable.

On CQA platforms few questions receive answers after a couple of days or even weeks. By that time, the seeker might already find the relevant information or is not active on the question. We analyze this further using the exploratory data analysis approach. We find that ~80% of the questions receive answers within a day or two, and the maximum time to receive the answer goes up to months. Thus, we included *days to answer* and measured it as the number of days between the time of the question and the time of the answer.

#### Model specification

Given our dependent variable–‘Success’ of helping–is a binary variable, we used hierarchical logistic regression analysis to examine our hypotheses [80]. We estimated our theoretical model based on three nested models. Next, we begin with a baseline model and add different factors on top of it in a stepwise manner [81]. This helps in isolating the influence of adding each factor on the model fit. Further, we standardized all continuous explanatory variables for a better interpretation of effect sizes and to minimize the issue of multicollinearity due to the polynomial and interaction terms in our model [82].

## 5. Results

Table 1 presents the descriptive statistics for all the explanatory variables we defined at different levels.

**Table 1:** Descriptive statistics of variables (*N = 72959*).

*Variable*	*VIF*	*Mean*	*S*.*D*.	*Min*	*Max*
Recent average reward gain	1.20	12.8	26.4	-10	500
Contribution frequency	2.49	5.8	9.3	1	81
Past accumulated experience	2.75	698.1	1495.4	1	7776
seeker’s status	1.00	314.6	2264.7	-50	30k
question length	1.02	109.2	90.1	0	1292
answer length	1.12	109.3	117	0	2032
number of external references (URLs)	1.10	0.93	1.65	0	10
days to answer	1.02	28.7	110.4	0	1068

The variation of inflation factors (VIF) of the independent and control variables ranges from 1.00 to 2.75, suggesting that multicollinearity issues among explanatory variables are within the acceptable range [82].

Table 2 presents the results of the hierarchical logistic regression model. We first start by adding the control variables in the baseline model (model with only intercept). In Model 2, we add the primary independent variables (i.e., reward gain and contribution frequency). In Model 3, we include the interaction terms. We conducted the likelihood ratio (LR) test to compare all the models, along with Akaike’s information criteria (AIC), and the Bayesian information criteria (BIC). We reject the null hypothesis at the 0.05 significance level for all the models. The significantly decreasing values of all three tests (LR, AIC, BIC) from Model 1 to Model 3, confirm the validity of our proposed model.

**Table 2:** Empirical results.

	*Model 1* *Baseline*	*Model 2+* *Main effect*	*Model 3+* *Interaction effect*
*Intercept*	*-1*.082*** (0.*012)*	*-1*.071*** (0.*012)*	*-1*.010*** (0.*015)*
***Main effect***			
Recent average reward gain		*0*.188*** (0.*009)*	*0*.145*** (0.*011)*
Contribution frequency		*0*.223*** (0.*020)*	*0*.149** (0.*033)*
Contribution frequency^2		*-0*.173*** (0.*020)*	*-0*.236** (0.*064)*
***Moderating effect***			
Past accumulated experience			*0*.335*** (0.*022)*
Recent reward gain * Past accumulated experience			*-0*.074*** (0.*009)*
Contribution frequency * Past accumulated experience			*-0*.152*** (0.*016)*
Contribution frequency ^2 * Past accumulated experience			*0*.110*** (0.*020)*
***Control***			
seeker’s status	*0*.025*** (0.*008)*	*0*.024*** (0.*008)*	*0*.023*** (0.*008)*
question length	*-0*.032*** (0.*009)*	*-0*.034*** (0.*009)*	*-0*.035*** (0.*009)*
answer length	*0*.081*** (0.*009)*	*0*.067*** (0.*008)*	*0*.062*** (0.*009)*
presence of code	*0*.459*** (0.*016)*	*0*.436*** (0.*016)*	*0*.449*** (0.*017)*
number of external references (URLs)	*0*.113*** (0.*009)*	*0*.097*** (0.*009)*	*0*.095*** (0.*009)*
days to answer	*-0*.608*** (0.*019)*	*-0*.541*** (0.*019)*	*-0*.521*** (0.*019)*
*Log-likelihood*	*-43672*	*-43302*	*-43182*
*X*^*2*^ *(model improvement* ^*#*^*)*		*739*.58***	*239*.79***
*AIC*	*87357*.*64*	*86624*.*06*	*86392*.*27*
*BIC*	*87422*.*02*	*86716*.*04*	*86521*.*04*
*N*	72959	72959	72959

Signif. codes: 0 ’***’ 0.001

’**’ 0.01

’*’ 0.05 ’.’ 0.1 ’ ’ 1

^#^Likelihood ratio test statistic

Model 2 in Table 2 shows, the positive effect of recent average reward gain is statistically significant (β = 0.188, p-value < 0.001). Therefore, hypothesis H1 is supported. This suggests that contributors who are rewarded on average higher for their past contributions put more effort and are more likely to provide ‘accepted’ answers. Further, the results show, that the positive effect of contribution frequency (answering more questions) is statistically significant (β = 0.223, p-value < 0.001) while the square term of contribution frequency measure is negatively and statistically significant (β = -0.173, p-value < 0.001). This suggests an inverted U-shaped relationship between contribution frequency and success. Thus, hypothesis H2 is supported.

Model 3 in Table 2 shows the interaction term of recent average gain in reward and past accumulated experience is negative and statistically significant (β = -0.074, p-value < 0.001). This suggests that past experience diminishes the positive effect of additional gain in reward points on the likelihood of success of the answer. In other words, novice contributors when receiving higher rewards put in more effort and provide more accurate answers. Accordingly, hypothesis H3a is supported.

**Fig 2:**
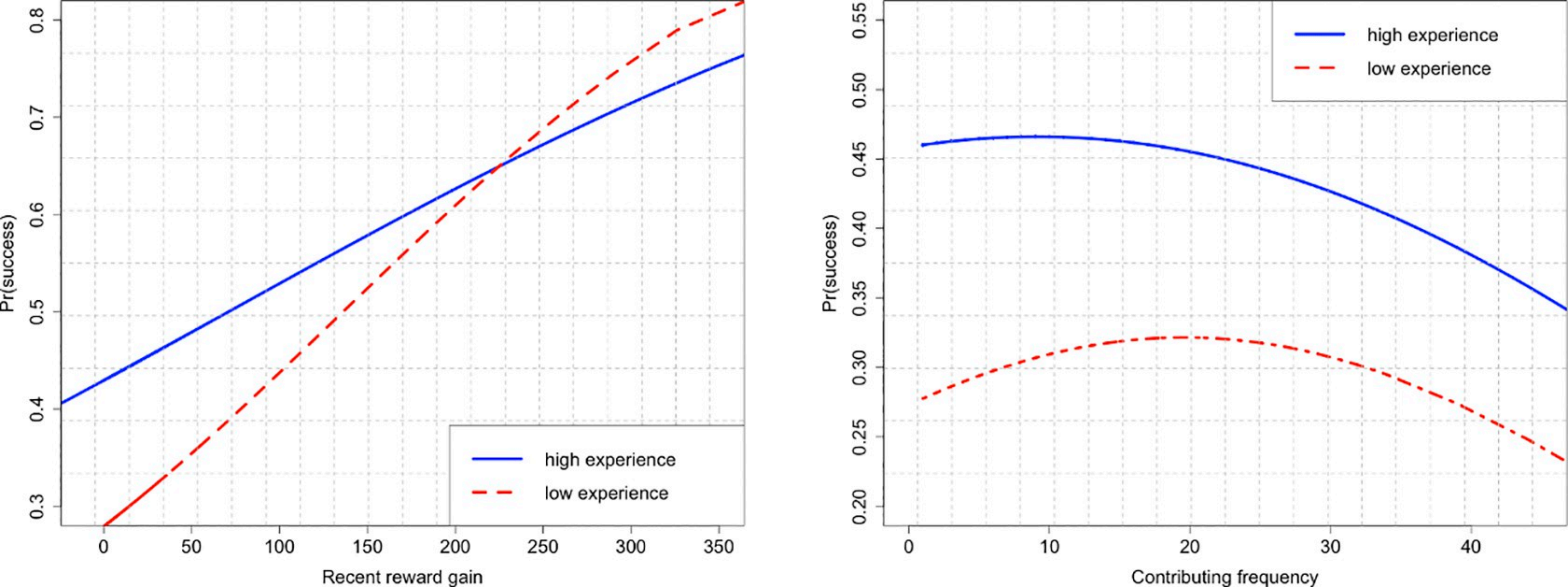
Illustrates the moderating role of past accumulated experience.

The interaction term of the contribution frequency and past accumulated experience is negative and statistically significant (β = -0.152, p-value < 0.001), while that between the contribution frequency square and past accumulated experience is positive and statistically significant (β = 0.110, p-value < 0.001). This suggests that learning dominates the crowding effect in the beginning for contributors with lesser experience. However, as learning is marginal for experienced contributors, the crowding-out effect is dominant. This shows that hypothesis H3b is supported. Fig 2 illustrates the moderating role of past accumulated experience.

### Robustness checks

In order to determine the robustness of the findings, we performed several additional analyses.

First, to assess the robustness check of our findings we use other similar CQA datasets to ‘Super User’ to eliminate potential bias from the dataset dependence. We test our hypothesis on two other CQA communities—‘Ask Ubuntu’ (https://askubuntu.com) and ‘Unix & Linux’ (https://unix.stackexchange.com). Both these communities are among the top four communities on the SE network based on the high traffic they receive (more than ~100k visits per day). ‘Ask Ubuntu’ is a focused community on topics related to Ubuntu users and developers. Similarly, ‘Unix & Linux’ is a Q&A community for users of Linus, FreeBSD, and other Unix-like operating systems. Both these communities started around the same time as the ‘Super User’ community and thus share a similar maturity level of the community. Following the settings as in our main analysis with the ‘Super User’ community, we use the most recent data (Jan 2017-Dec 2019) for the robustness checks, and filtering the questions that did not receive any answer from the dataset. The results suggested that our findings are robust to similar alternative communities and can be generalized (see SI Table S1 and S2 in S1 File).

Second, given our analysis relies on observational panel data, there exists the possibility of unobserved individual characteristics that could potentially impact the final results. In particular, the success of knowledge contribution may be influenced by contributors’ domain knowledge. Therefore, we introduced additional measure to consider individual expertise as a control variable. Prior research has emphasized that users’ expertise tends to evolve on CQA platforms [83, 84]. Acknowledging the limitations of observational data in directly measuring expertise or domain knowledge, we utilize badges earned by individuals prior to answer contribution as a proxy. On Stack Exchange platforms, contributors receive badges in recognition of outstanding knowledge contribution. Hence, we employ the count of answer-gold badges (the highest level of badges) awarded to contributors up until the time of answering as an indicator of their expertise level. Considering the nature of our dataset, we employ a hierarchical logistic regression approach that allows for unbalanced panel data [81]. In our case, multiple observations are available for each individual, and the number of observations varies among individuals. We implemented a mixed-effect logistic regression model, that enable us to control for level-specific unobserved heterogeneity by incorporating random-effect terms capturing unobserved individual characteristics. This aligns with prior studies in similar research contexts [12] and ensures precise estimates of measures by accounting for unobserved variations at the individual levels. The results of the analysis are included in S3 Table in S1 File (see SI). The consistency of the results in the series of tests suggests the robustness of our results.

Third, we apply an alternative measure of answer quality as our DV. Existing studies within the realm of OCs have frequently employed the count of helpful votes on a post as an indicator of quality. While the designation of the best answer implies seeker satisfaction, the number of votes potentially imparts distinct information about how widely perceived and helpful the community members find it, effectively functioning as a measure of popularity along with quality. This metric is thus designed to capture the broader community’s acknowledgment of an answer. It is important to consider that answers posted early may receive more votes from community participants. To mitigate the potential bias introduced by this time effect, we restricted the consideration of votes received by each answer to a one-year time window from its posting date. Given the DV, the number of votes received is a count variable with different mean and variance, we employed the negative binomial regression [85]. One notable advantage of negative binomial regression models is their accommodation of unequal mean and variance, introducing a parameter to address potential over-dispersion in the data. Notably, the results obtained with the new DV shows positive effect of extrinsic reward, indicating that contributors who have received higher previous positive reward put efforts resulting in an increase in the perceived quality and popularity of their answers (see SI Table S4 in S1 File). Conversely, an inflated contribution frequency exerts a negative effect on the number of votes received by the answers. Intriguingly, while no inverted-U relationship is observed between the new DV and contribution frequency, the significant negative effect underscores the adverse effect of inflated contribution on the perceived quality and popularity of answers. This highlights the presence of crowding-out effect, albeit with variations in the nature of crowding-effect between the two DV measures. One plausible explanation for this discrepancy could be the distinct scrutiny applied by the crowd in assessing answers through votes, considering additional details and presentation factors that impact their appeal and popularity. Furthermore, the moderating effect of the contributors’ experience was not significant for the new DV. Given the accepted answer measure and number of votes are inherently distinct DVs, the results are expected differently. This opens a captivating avenue for future research to delve into how crowding-out effect manifests differently for measures that amalgamate popularity and crowd-perceived helpfulness.

## 6. Discussion and conclusion

Online question-and-answer communities are becoming increasingly popular, changing the way people share and seek knowledge. While these platforms offer significant help to knowledge seekers, a key challenge they face, amid the rapid growth, is to ensure high-quality contribution that addresses seekers’ specific information needs [86]. Motivated by the importance of the success of answer contribution in this context and the gap in the literature, this study sought to investigate the factors that shape successful helping in CQAs. Specifically, this research draws upon self-determination theory and motivation crowding literature to investigate the contributing behavior that led to success. An observational panel dataset collected from a large CQA website was used and several interesting findings were derived from this study. We find that the reward system positively influences the success of the contribution. However, while extant research highlights the influence of external rewards on the quantity of contribution, we find that the inflated frequency of contribution led to the crowding-out effect. In particular, we find that the contribution frequency has a curvilinear relationship with the success of the contribution. Interestingly, users’ past accumulated experience moderates the effect of additional rewards as well as the impact of high contribution frequency.

This study makes important theoretical implications for the online Q&A community literature as well as the motivation crowding theory. This study advances our understanding of the success of knowledge contribution in online Q&A communities. While most of the extant research in CQA, and OC in general, focuses on the quantity aspect of knowledge contribution [14, 20, 54], our work highlights the contributing behavior that impacts the success of knowledge contribution in a utilitarian online knowledge community. The empirical results reveal the significance of gain in rewards in promoting quality contribution and confirm the general information systems design principle that timely positive feedback needs to be provided to satisfy users’ need for competence [87].

Next, our study contributes significantly to the literature on incentives and motivation crowding. Extant literature has examined the role of monetary incentives to encourage, and promote pro-social activities and highlighted that these incentives result in a crowding-out effect. This study complements the literature on the crowding-out effect, studying it in online Q&A communities governed by symbolic incentive mechanisms. While past studies have highlighted the role of external rewards in promoting high-quantity contribution in OCs, the findings in this study provided evidence pointing to the presence of the crowding-out effect resulting from the inflated frequency of contributions.

Further, this research shows that contributors’ past accumulated experience moderates the impact of additional rewards and contribution frequency. Specifically, our findings show that the positive effect of additional reward gain is less effective for experienced contributors. Also, higher past experience shifts the inverted U-shaped relationship between the contribution frequency and the success of the contribution. This can be explained by the learning effect of contribution which states that users learn when they contribute more. For inexperienced users, the initial increase in contribution results in learning and thus increases the likelihood of success. However, for experienced users, the learning is marginal, and thus the crowding-out effect dominates, decreasing the likelihood of success as the contribution frequency increases. This raises an important question for future research on how to better motivate community members so as to engage in successful knowledge contribution.

### Practical implications

Besides its theoretical implications, this study holds significant practical implications for platform managers and developers desiring to encourage quality knowledge sharing on the platforms.

First, this study reaffirms the significance of a design principle in information systems, emphasizing that extrinsic rewards are essential to motivate users to contribute knowledge to the platform [87]. However, a crucial challenge for designers lies in striking a balance between intrinsic and extrinsic motivations to encourage and motivate contributors effectively. While the designers of online knowledge-sharing platforms might assume an additive effect of intrinsic and extrinsic motivation, the hidden costs of extrinsic motivation must be considered. If the controlling effect of extrinsic motivation dominates, contributors may conform to the principle of direct reciprocity by contributing knowledge solely for rewards. For instance, contributors in our study on Stack Exchange platforms receive 10 reward points for every positive vote on their answers and an additional 15 reward points if their answer is accepted by the seeker. Consequently, contributors highly motivated by external reward points may not invest extra effort and time in providing tailored answers that more precisely address the seeker’s specific information needs. Instead, their focus might shift towards answering more questions that might still fetch them votes from other members on the platform.

Therefore, our study underscores the necessity for practitioners of online communities to reconsider or redesign gamification mechanisms to better motivate voluntary knowledge contribution. Managers of Q&A sites and OCs in general, should innovate gamification mechanisms with a focus on participants’ intrinsic motivation, which is closely linked to their higher-order needs. For example, instead of simply increasing rewards for accepted answers, managers could encourage designs that promote the intrinsic motivation of learning and enjoyment derived from sharing knowledge. Providing more opportunities for contributors to receive positive symbolic feedback in the form of appreciation, such as a thank you note from seekers is one such approach. This can be done by prompting seekers to express gratitude for receiving answers, fostering a culture of recognition for informative knowledge contributors.

Furthermore, our study reveals that while active engagement on platforms fosters a learning effect, an inflated contribution pattern of posting an excessive number of answers in a short span of time negatively impacts quality. Online platforms receive thousands of queries every day. Thus, platform managers should also ensure that contributors are not overwhelmed with an excessive volume of questions. An effective approach is to identify all potential active contributors and recommend newly posted questions evenly to them. Designers and managers can also leverage advancements in unbiased machine learning algorithms to ensure a fair and balanced routing of questions.

Lastly, our study underscores that the positive impact of rewards is more pronounced for novice users compared to experienced users. Consequently, managers of OCs should tailor the motivational mechanisms with consideration of contributors’ experience levels. For novice users, a reward system can serve as a highly effective motivator, fostering a sense of helpfulness and community engagement. This approach can be particularly beneficial for newly established OCs, where members can be externally motivated initially through external reward points. However, it is crucial for managers to acknowledge that excessive reliance on external rewards may eventually diminish the intrinsic enjoyment derived from helping others. Therefore, as members become more active in these OCs, the emphasis on rewards should be reduced, and the promotion of symbolic recognition systems should be encouraged.

In essence, a well-designed incentive system for user-generated content (UGC) communities like Q&A forums (like Stack Exchange, Yahoo Answers), review sites (like TripAdvisor, Amazon), discussion forums (such as Reddit, Quora), should primarily focus on cultivating and preserving the intrinsic motivations of its members. If supplementary motivation is considered necessary, the incorporation of extrinsic rewards should be implemented in a manner that does not impede or undermine these intrinsic motivations.

### Limitations and future research directions

While this study possesses notable strengths, it is not without limitations, thereby opening avenues for future research.

First, our findings are grounded in a specific type of OCs, that is community question-answering websites, predominantly focusing on IT topics. The generalizability of these findings may be constrained when applied to other technical Q&A communities where users’ domain knowledge is crucial for making quality contributions, such as healthcare Q&A communities or organizational enterprise systems. However, the applicability of these findings to different forms of OCs, characterized by distinct user populations, IT artifacts, topic characteristics, and organizational structure (e.g., online customer review sites, social commerce sites) remain uncertain, Unlike the technical-focused platforms, the platforms that are opinion based usually do not necessitate specific subject knowledge for providing high quality answers. It would be valuable to extend the current study by conducting similar investigations across diverse sites, including online social Q&A platforms like Quora, collaborative platforms like Wikipedia, e-commerce and review sites such as Amazon, and user-generated content sites in general.

The second limitation is associated with the study’s design. Our analysis relies on extensive observational panel datasets, that are capable of depicting individual behavior with minimal subjective bias. The unique advantages of user-generated datasets, which enable rigorous testing and the exploration of various levels of habitual behavior, have been acknowledged in recent studies [8, 88]. However, while this type of analysis offers valuable evidence regarding the theoretical relationships among the variables of interest, it might not be sufficient for establishing the underlying causality grounded in theories. Future research endeavors could enhance causal identification by incorporating randomized field experiments into their methodology.

Furthermore, it would be valuable to investigate knowledge-contributing behavior on CQAs that serve as a recognizable signal for potential employers can be investigated. Contributions in technical OCs like Github are already used by contributors to signal their technical expertise while approaching a potential employer. It would be interesting to analyze the difference in contributing behavior of contributors who participate based on only their interest in the topic and pro-social behavior with those who participate to potentially enter an industry related to the field discussed in CQA.

## Supporting information

S1 File Contains the following files: Robustness checks results—Tables: S1 Empirical results–Ask Ubuntu CQA community (2). S2 Empirical results–Unix & Linux CQA community (3). S3 Empirical results–SuperUser CQA community–Mixed-effect logistic regression (4). S4 Empirical results–SuperUser CQA community (*DV-Answer Vote Score; Model—Mixed Effect Negative Binomial Regression*) (5).
(PDF)
