# Supplementary material for: “The double-edged sword of inflated help”: Unravelling the motivation crowding in community question-answering platforms
Source: PLoS One. 2024 Mar 13;19(3):e0297627. doi: 10.1371/journal.pone.0297627 (PMC10936805; doi:10.1371/journal.pone.0297627)
Supplement: S1 File — S2 Empirical results–Unix & Linux CQA community (3). S3 Empirical results–SuperUser CQA community–Mixed-effect logistic regression (4). S4 Empirical results–SuperUser CQA community (DV-Answer Vote Score; Model—Mixed Effect Negative Binomial Regression) (5). (PDF) [file pone.0297627.s001.pdf]

Supporting Information for

"The Double-Edged Sword of Inflated Help": Unravelling the Motivation Crowding in Community Question-Answering Platforms

Vaibhav Krishna[1,2] *, Yash Raj Shrestha[3], Georg von Krogh[4,5]

[1] Yale Institute of Network Science, Yale University, New Haven, Connecticut, United States of America

[2] Department of Sociology, Yale University, New Haven, Connecticut, United States of America

[3] Faculty of Business and Economics (HEC), University of Lausanne, Lausanne, Switzerland

[4] Department of Management, Technology and Economics, ETH Zürich, Zürich, Switzerland

[5] ETH AI Center, ETH Zürich, Zürich, Switzerland

*Corresponding author

Email: vaibhav.krishna@yale.edu

Contents

Robustness checks results

Table S1: Empirical results – Ask Ubuntu CQA community 2

Table S2: Empirical results – Unix & Linux CQA community 3

Table S3: Empirical results – SuperUser CQA community – Mixed-effect logistic regression 4

Table S4: Empirical results – SuperUser CQA community
(*DV-Answer Vote Score; Model - Mixed Effect Negative Binomial Regression*) 5

Table S1: Empirical results – Ask Ubuntu CQA community

	Model 1 Baseline	*Model 2+ Main effect*	*Model 3+ Moderation effect*
Intercept	*-1.405*** (0.019)*	*-1.328*** (0.019)*	*-1.215*** (0.020)*
Main effect			
Recent average reward gain		*0.230*** (0.009)*	*0.275*** (0.013)*
Contribution frequency		*0.322*** (0.021)*	*0.208*** (0.023)*
Contribution frequency ^2		*-0.233*** (0.022)*	*-0.146*** (0.022)*
Moderating effect			
Past accumulated experience			*0.203*** (0.017)*
Recent average reward gain * Past accumulated experience			*-0.096*** (0.006)*
Contribution frequency * Past accumulated experience			*-0.151*** (0.028)*
Contribution frequency ^2 * Past accumulated experience			*0.078** (0.030)*
Control			
seeker's status	*0.057*** (0.010)*	*0.048*** (0.009)*	*0.047*** (0.009)*
question length	*-0.007 (0.009)*	*0.009 (0.009)*	*-0.009 (0.009)*
answer length	*0.058*** (0.0120)*	*0.034*** (0.010)*	*0.024* (0.010)*
presence of code	*0.538*** (0.021)*	*0.438*** (0.021)*	*0.405*** (0.021)*
number of external references (URLs)	*0.072*** (0.010)*	*0.047*** (0.010)*	*0.045*** (0.010)*
days to answer	*-0.906*** (0.025)*	*-0.752*** (0.024)*	*-0.712*** (0.024)*
Log-likelihood	*-36349*	*-35818*	*-35650*
X^2 (model improvement [#])		*1062.58****	*335.62****
AIC	*72711.59*	*71655.01*	*71327.39*
BIC	*72774.97*	*71745.55*	*71454.15*
N	*63201*	*63201*	*63201*

*Signif. codes: 0 '***' 0.001 '**' 0.01 '*' 0.05 '.' 0.1 ' ' 1*
[#]Likelihood ratio test statistic

Table S2: Empirical results – Unix & Linux CQA community

	Model 1 Baseline	*Model 2+ Main effect*	*Model 3+ Moderation effect*
Intercept	*-1.020*** (0.025)*	*-0.926*** (0.025)*	*-0.828*** (0.026)*
Main effect			
Recent average reward gain		*0.196*** (0.013)*	*0.332*** (0.021)*
Contribution frequency		*0.360*** (0.020)*	*0.223*** (0.024)*
Contribution frequency ^2		*-0.242*** (0.020)*	*-0.138*** (0.025)*
Moderating effect			
Past accumulated experience			*0.166*** (0.019)*
Recent average reward gain * Past accumulated experience			*-0.072*** (0.004)*
Contribution frequency * Past accumulated experience			*-0.110*** (0.025)*
Contribution frequency ^2 * Past accumulated experience			*0.052* (0.026)*
Control			
seeker's status	*0.006 (0.008)*	*-0.004 (0.008)*	*-0.007 (0.008)*
question length	*0.021* (0.008)*	*0.039*** (0.008)*	*0.041*** (0.008)*
answer length	*0.162*** (0.009)*	*0.123*** (0.009)*	*0.117*** (0.009)*
presence of code	*0.434*** (0.026)*	*0.324*** (0.027)*	*0.288*** (0.027)*
number of external references (URLs)	*0.115*** (0.009)*	*0.111*** (0.009)*	*0.109*** (0.009)*
days to answer	*-0.464*** (0.017)*	*-0.407*** (0.017)*	*-0.390*** (0.017)*
Log-likelihood	*-44165*	*-43721*	*-43561*
X^2 (model improvement #)		*886.85****	*321.04****
AIC	*88343.70*	*87462.85*	*87149.81*
BIC	*88407.78*	*87554.39*	*87277.97*
N	*69839*	*69839*	*69839*

*Signif. codes: 0 '***' 0.001 '**' 0.01 '*' 0.05 '.' 0.1 ' ' 1*
#Likelihood ratio test statistic

Table S3: Empirical results – SuperUser CQA community – Mixed-effect logistic regression

	Model 1 Baseline	*Model 2+ Main effect*	*Model 3+ Moderation effect*
Intercept	*-1.202*** (0.023)*	*-1.19*** (0.024)*	*-1.136*** (0.031)*
Main effect			
Recent average reward gain		*0.027** (0.010)*	*0.027** (0.010)*
Contribution frequency		*0.061* (0.030)*	*0.125** (0.041)*
Contribution frequency ^2		*-0.052* (0.024)*	*-0.154* (0.073)*
Moderating effect			
Past accumulated experience			*0.101 (0.070)*
Recent average reward gain * Past accumulated experience			*-0.027* (0.010)*
Contribution frequency * Past accumulated experience			*-0.084*** (0.021)*
Contribution frequency ^2 * Past accumulated experience			*0.068** (0.023)*
Control			
seeker's status	*0.017* (0.008)*	*0.017* (0.008)*	*0.017* (0.008)*
question length	*-0.022* (0.009)*	*-0.022* (0.009)*	*-0.022* (0.009)*
answer length	*0.050*** (0.010)*	*0.050*** (0.010)*	*0.050*** (0.010)*
presence of code	*0.333*** (0.019)*	*0.333*** (0.019)*	*0.333*** (0.019)*
number of external references (URLs)	*0.088*** (0.010)*	*0.088*** (0.010)*	*0.087*** (0.010)*
days to answer	*-0.487*** (0.019)*	*-0.484*** (0.019)*	*-0.478*** (0.019)*
contributor expertise	*0.276*** (0.025)*	*0.264*** (0.025)*	*0.260** (0.035)*
contributor random-effect	*yes*	*yes*	*yes*
Log-likelihood	*-42366*	*-42360*	*-42350*
X^2 (model improvement #)		*12.47****	*20.22****
AIC	*84750.40*	*84743.93*	*84731.72*
BIC	*84833.18*	*84854.30*	*84878.88*
N	*72959*	*72959*	*72959*

*Signif. codes: 0 '***' 0.001 '**' 0.01 '*' 0.05 '.' 0.1 ' ' 1*
#Likelihood ratio test statistic

Table S4: Empirical results – SuperUser CQA community
DV-Answer Vote Score; Model - Mixed Effect Negative Binomial Regression

	Model 1 Baseline	*Model 2+ Main effect*	*Model 3+ Moderation effect*
Intercept	*-0.039*** (0.013)*	*-0. 044*** (0.011)*	*-0.045*** (0.017)*
Main effect			
Recent average reward gain		*0.011** (0.004)*	*0.010* (0.005)*
Contribution frequency		*-0.069*** (0.015)*	*-0.035** (0.012)*
Contribution frequency ^2		*0.021 (0.012)*	
Moderating effect			
Past accumulated experience			*-0.062* (0.032)*
Recent average reward gain * Past accumulated experience			*0.002 (0.004)*
Contribution frequency * Past accumulated experience			*-0.002 (0.004)*
Control			
seeker's status	*0.028*** (0.003)*	*0.028*** (0.003)*	*0.028*** (0.003)*
question length	*-0.036*** (0.004)*	*-0.037*** (0.004)*	*-0.037*** (0.004)*
answer length	*0.060*** (0.004)*	*0.060*** (0.004)*	*0.060*** (0.004)*
presence of code	*0.047*** (0.004)*	*0.047*** (0.005)*	*0.047*** (0.005)*
number of external references (URLs)	*0.065*** (0.004)*	*0.065*** (0.004)*	*0.065*** (0.004)*
days to answer	*-0.054*** (0.005)*	*-0.054*** (0.005)*	*-0.054*** (0.005)*
contributor expertise	*0.053*** (0.015)*	*0.072*** (0.015)*	*0.101*** (0.019)*
contributor random-effect	*yes*	*yes*	*yes*
Log-likelihood	*-99814*	*-99784*	*-99782*
X^2 (model improvement [#])		*59.84****	*3.41*
AIC	*199647.0*	*199593.2*	*199593.8*
BIC	*199738.3*	*199711.9*	*199730.7*
N	*68253*	*68253*	*68253*

*Signif. codes: 0 '***' 0.001 '**' 0.01 '*' 0.05 '.' 0.1 ' ' 1*
[#]Likelihood ratio test statistic

DV: number of votes received by each answer in one year time window from its posting
We consider the potential impact of outlier noise in DV, leading to significant skewness in our dataset. To mitigate this potential bias, we exclude observations with outlier values in the DV. Subsequently, the skewness decreases from 18.43 to 1.17
